# 成人血液病患者异基因造血干细胞移植后呼吸道合胞病毒感染的临床特征及死亡相关危险因素分析

**DOI:** 10.3760/cma.j.cn121090-20240424-00162

**Published:** 2024-10

**Authors:** 瑶 李, 枫 张, 畅 刘, 晓甦 赵, 晓冬 莫, 峰蓉 王, 晨华 闫, 志东 王, 军 孔, 圆圆 张, 凤美 郑, 扬 刘, 乐清 曹, 道兴 邓, 晓军 黄, 晓辉 张

**Affiliations:** 1 北京大学人民医院血液科，北京大学血液病研究所，国家血液系统疾病临床医学研究中心，造血干细胞移植治疗血液病北京市重点实验室，北京 100044 Peking University People's Hospital, Peking University Institute of Hematology, National Clinical Research Center for Hematologic Disease, Beijing Key Laboratory of Hematopoietic Stem Cell Transplantation, Peking-Tsinghua Center for Life Science, Research Unit of Key Technique for Diagnosis and Treatment of Hematologic Malignancies, Chinese Academic of Medical Sciences, Beijing 100044, China; 2 北京大学人民医院检验科，北京 100044 Department of Clinical Laboratory, Peking University People's Hospital, Beijing 100044, China

**Keywords:** 造血干细胞移植, 呼吸道合胞病毒, 移植物抗宿主病, 免疫抑制剂, Hematopoietic stem cell transplantation, Respiratory syncytial virus, RSV, Graft-versus-host disease, Immunosuppressants

## Abstract

**目的:**

探究成人血液病患者异基因造血干细胞移植（allo-HSCT）后呼吸道合胞病毒（RSV）感染的临床特征及死亡相关危险因素。

**方法:**

纳入2023年10月至2024年1月期间在北京大学人民医院血液科检出RSV阳性的allo-HSCT患者，对其临床资料进行回顾性分析，采用二元Logistic回归分析死亡相关危险因素。

**结果:**

共纳入移植后RSV检测阳性患者43例，男26例，女17例，中位年龄42（21～66）岁。急性髓系白血病25例，急性淋巴细胞白血病9例，骨髓增生异常综合征6例，再生障碍性贫血1例，淋巴瘤1例，混合性白血病1例。单倍体移植33例，同胞全相合移植8例，无关供者移植2例。20例（46.5％）患者因RSV感染或合并其他感染发生低氧血症，6例（14.0％）转ICU治疗，4例（9.3％）气管插管辅助通气，7例（16.3％）患者死亡。死亡患者感染RSV时PLT较低［15.0（10.0～62.0）×10^9^/L对74.5（8.0～348.0）×10^9^/L，*P*＝0.003］，合并细菌感染比例较高（85.7％对41.7％，*P*＝0.046），血液学复发比例较高（71.4％对13.9％，*P*＝0.004）。血液学复发（*OR*＝15.500，95％*CI* 2.336～102.848，*P*＝0.005）、甲型流感病毒感染（*OR*＝14.000，95％*CI* 1.064～184.182，*P*＝0.045）、感染RSV时PLT较低（*OR*＝0.945，95％*CI* 0.894～0.999，*P*＝0.048）是移植后患者死亡的危险因素。

**结论:**

allo-HSCT后感染RSV的成人血液病患者预后不良。

## Abstract (en)

**Objective:**

To summarize the clinical features associated with respiratory syncytial virus（RSV）infection in patients following the hematopoietic stem cell transplant（HSCT）and exploring the risk factors for death.

**Methods:**

Patients who had RSV infection after undergoing HSCT from October 2023 to January 2024 in the hematology department of Peking University People's Hospital were enrolled in the study. The clinical characteristics of the participating patients were summarized. The clinical characteristics of the surviving and the dying patients were compared, and the risk factors of death were analyzed by binary logistic regression.

**Results:**

Among the 43 RSV-positive HSCT patients, 20（46.5%）were hypoxemic, six（14.0%）were admitted to the ICU for further treatment, four（9.3%）required tracheal intubation assisted ventilation, and seven patients（16.3%）died. A comparison of the clinical features of the surviving patients and the deceased patients demonstrated that the deceased patients had a lower PLT when infected with RSV［74.5（8.0–348.0）×10^9^/L *vs* 15.0（10.0–62.0）×10^9^/L, *P*＝0.003］, a higher incidence of simultaneous bacterial infections（85.7% *vs* 41.7%, *P*＝0.046）, and a higher rate of hematological recurrence（71.4% *vs* 13.9%, *P*＝0.004）. Hematological recurrence（*OR*＝15.500, 95% *CI* 2.336–102.848, *P*＝0.005）, influenza A viral infection（*OR*＝14.000, 95%*CI* 1.064–184.182, *P*＝0.045）, and low PLT at the time of RSV infection（*OR*＝0.945, 95% *CI* 0.894–0.999, *P*＝0.048）were the factors associated with death following HSCT.

**Conclusion:**

Patients infected with RSV after undergoing HSCT have a poor prognosis, and active prevention and treatment of RSV in the autumn and winter requires urgent attention.

呼吸道合胞病毒（RSV）是秋、冬、春季高发的呼吸道病原体，在造血干细胞移植（HSCT）患者中发病率为2％～35％[1]–[3]，可引起病毒性肺炎并与高病死率有关[4]。据报道，RSV是HSCT患者中最常见的季节性呼吸道病毒感染病原[5]–[6]，占呼吸道病毒感染的30％～37％，其中19％～36％为下呼吸道感染（LRTI）[7]。与其他呼吸道病原相比，RSV感染引起的肺炎发病率和病死率更高[8]，儿童病死率<5％[9]，成人病死率约10％[10]。目前RSV的治疗选择有限，缺乏大样本队列研究资料，国内关于HSCT患者感染RSV的报道较少。本研究对我院并发RSV感染的异基因造血干细胞移植（allo-HSCT）患者进行回顾性分析，探究RSV感染的临床特征及死亡相关因素。

## 病例与方法

一、研究对象

2023年10月至2024年1月期间，280例在北京大学人民医院血液科治疗的成年血液病患者接受呼吸道病原检测，其中50例（17.86％）RSV检测阳性，43例为allo-HSCT后患者。收集allo-HSCT后患者的临床资料（性别、年龄、诊断、移植情况、混合感染情况、重症感染情况等），回顾性分析其临床特征、治疗及预后。

二、预处理及移植方案

所有患者均采用我中心常规预处理方案。

33例单倍体移植患者：①改良白消安（Bu）/环磷酰胺（Cy）联合兔抗人胸腺细胞免疫球蛋白（rATG）清髓预处理方案：阿糖胞苷（Ara-C）4 g/m^2^静脉滴注，−9 d；Bu 0.8 mg/kg每6 h 1次静脉滴注，−8 d～−6 d；Cy 1.8 g·m^−2^·d^−1^静脉滴注，−5 d、−4 d；rATG 2.5 mg·kg^−1^·d^−1^，−5 d～−2 d；司莫司汀250 mg/m^2^口服，−3 d。②Bu/Cy/氟达拉滨（Flu）+rATG方案：Ara-C 4 g/m^2^静脉滴注，−9 d；Bu 0.8 mg/kg每6 h 1次静脉滴注，−8 d～−6 d；Flu 30 mg·m^−2^·d^−1^静脉滴注，−6 d～−2 d；Cy 1.0 g·m^−2^·d^−1^静脉滴注，−5 d、−4 d；rATG 2.5 mg·kg^−1^·d^−1^，−5 d～−2 d；司莫司汀250 mg/m^2^口服，−3 d。③地西他滨（DAC）+Bu/Cy+rATG：DAC 200 mg·m^−2^·d^−1^静脉滴注，−12 d、−11 d；Ara-C 4 g/m^2^静脉滴注，−10 d、−9 d；Bu 0.8 mg/kg每6 h 1次静脉滴注，−8 d～−6 d；Cy 1.8 g·m^−2^·d^−1^静脉滴注，−5 d、−4 d；rATG 2.5 mg·kg^−1^·d^−1^，−5 d～−2 d；司莫司汀250 mg/m^2^口服，−3 d。

8例同胞全相合移植患者：①改良Bu/Cy方案：Ara-C 4 g/m^2^静脉滴注，−9 d；Bu 0.8 mg/kg每6 h 1次静脉滴注，−8 d～−6 d；Cy 1.8 g·m^−2^·d^−1^静脉滴注，−5 d、−4 d；司莫司汀250 mg/m^2^口服，−3 d，②改良Bu/Flu方案：Ara-C 2 g/m^2^静脉滴注，−9 d；Bu 0.8 mg/kg每6 h 1次静脉滴注，−8 d～−6 d；Flu 30 mg·m^−2^·d^−1^静脉滴注，−6 d～−2 d；司莫司汀250 mg/m^2^口服，−3 d，③全身放射治疗（TBI）/Cy方案：TBI 770 cGy，−6 d；Cy 1.8 g·m^−2^·d^−1^静脉滴注，−5 d、−4 d；司莫司汀250 mg/m^2^口服，−3 d。其中5例给予rATG 1.5 mg·kg^−1^·d^−1^，−4 d～−2 d。

所有供者常规行粒细胞集落刺激因子（G-CSF）5 µg·kg^−1^·d^−1^动员骨髓和（或）外周血造血干细胞，于用药第4～5天开始采集骨髓和（或）外周血造血干细胞并经中心静脉回输。粒细胞植入标准为中性粒细胞绝对计数（ANC）≥0.5×10^9^/L连续3 d且脱离G-CSF支持；血小板植入标准为PLT≥20×10^9^/L连续7 d且脱离血小板输注。血小板重建不良定义为移植后60 d粒系及红系重建良好而PLT<50×10^9^/L。

GVHD预防方案为环孢素A（CsA）、霉酚酸酯（MMF）联合短程甲氨蝶呤（MTX）。

三、病原体检测

使用一次性植绒拭子收集咽部标本，并保存在细胞保存液内。通过RT-PCR处理标本，对呼吸道病原体进行检测，包括甲型流感病毒、乙型流感病毒、RSV、鼻病毒、腺病毒、肺炎支原体。

针对检测病原体核酸保守区设计的特异性引物、特异荧光探针，配以PCR反应液等组分，在荧光定量PCR仪上，应用多重实时荧光定量PCR检测技术，通过荧光信号的变化实现对样本中的呼吸道病原体核酸的检测。PCR检测体系中含有阳性内对照，实验过程中通过检测样本中编码甘油醛-3-磷酸脱氢酶的人管家基因是否正常扩增来监测待测样本提取过程和PCR扩增过程，避免假阴性结果。

细菌和真菌检测主要通过患者痰液、支气管灌洗液、血液培养及药敏结果获得。

四、定义

上呼吸道感染（URTI）定义为在上呼吸道标本中检出RSV并咽痛、鼻塞、流涕等症状且不符合LRTI标准。

LRTI定义为存在以下任何一种情况：咳嗽伴咳痰、呼吸困难、胸痛或不适、低氧血症或肺部浸润影。

五、RSV的治疗

目前尚无成人RSV感染相关临床诊治指南或专家共识，参考儿童呼吸道合胞病毒感染临床诊治中国专家共识[11]和相关文献[12]–[13]，治疗包括抗病毒治疗、支持治疗。抗病毒治疗主要以利巴韦林为主，包括静脉、口服、雾化等不同的给药方式；支持治疗包括呼吸支持改善通气、液体管理等，其他治疗包括静脉注射免疫球蛋白、糖皮质激素等。

六、统计学处理

应用SPSS 22.0软件进行数据分析。对所有患者临床信息进行描述性分析，非正态分布的连续变量采用*U*检验，分类变量采用*χ*^2^检验，采用单因素Cox分析全因死亡的相关因素。*P*<0.05为差异具有统计学意义。

## 结果

一、临床基本资料

本研究共纳入移植后RSV检测阳性患者43例，其中男26例，女17例，中位年龄42（21～66）岁。急性髓系白血病（AML）25例，急性淋巴细胞白血病（ALL）9例，骨髓增生异常综合征（MDS）6例，再生障碍性贫血（AA）1例，淋巴瘤1例，混合性白血病1例。单倍体移植33例，同胞全相合移植8例，无关供者移植2例。有核细胞（NC）中位回输量为9.75（5.90～17.50）×10^8^/kg，CD34^+^细胞中位回输量为3.38（1.12～10.47）×10^6^/kg。粒细胞中位植入时间为13（9～21）d，血小板中位植入时间为13（8～42）d。感染RSV时疾病状态：完全缓解27例、血液学复发10例、分子生物学复发6例。感染RSV时7例患者合并急性GVHD，7例合并慢性GVHD。患者一般临床资料情况见表1。

**表1:** 43例异基因造血干细胞移植后呼吸道合胞病毒（RSV）感染患者一般临床资料

临床资料	结果
性别［例（%）］	
男	26（60.5）
女	17（39.5）
年龄［岁，*M*（范围）］	42（21~66）
造血干细胞移植时年龄［岁，*M*（范围）］	40（21~66）
临床诊断［例（%）］	
急性髓系白血病	25（58.1）
急性淋巴细胞白血病+淋巴瘤	9（20.9）
急性混合型白血病	1（2.3）
骨髓增生异常综合征	6（14.0）
再生障碍性贫血	1（2.3）
淋巴瘤	1（2.3）
HCT-CI评分［例（%）］	
0分	29（67.4）
1~2分	9（20.9）
≥3分	5（11.6）
供患者血型［例（%）］	
相合	19（44.2）
主要不合	12（27.9）
次要不合	9（20.9）
主要不合+次要不合	3（7.0）
供者类型［例（%）］	
单倍体	33（76.7）
同胞全相合	8（18.6）
非血缘供者	2（4.7）
干细胞来源	
外周血	40（93.0）
骨髓+外周血	1（2.3）
骨髓+外周血+脐带血	2（4.7）
外周血有核细胞［×10^8^/kg，*M*（范围）］	9.75（5.90~17.50）
外周血CD34^+^细胞［×10^6^/kg，*M*（范围）］	3.38（1.12~10.47）
粒细胞植入时间［d，*M*（范围）］	13（9~21）
血小板植入时间［d，*M*（范围）］	13（8~42）
感染RSV时疾病状态［例（%）］	
完全缓解	27（62.8）
血液学复发	10（23.3）
感染RSV时急性GVHD［例（%）］	
无	36（83.7）
Ⅰ/Ⅱ度	7（16.3）
Ⅲ/Ⅳ度	0（0）
感染RSV时慢性GVHD［例（%）］	
无	36（83.7）
轻度	3（7.0）
中度	3（7.0）
重度	1（2.3）
感染RSV时分子生物学复发［例（%）］	6（14.0）

**注** HCT-CI：造血干细胞移植合并症指数；GVHD：移植物抗宿主病

二、RSV感染和治疗

RSV感染的中位发生时间为移植后150（0～2 121）d。17例患者RSV感染发生于移植后100 d内。发生RSV感染时所有患者均获得粒细胞植入，4例患者未获得血小板植入。所有患者均有不同程度的临床表现，主要症状包括鼻涕、咳嗽、咳痰、头痛、咽痛、发热、寒战、呼吸困难等。25例患者影像学表现为肺浸润灶。

16例患者未合并其他病原体感染，27例（62.8％）患者发生混合感染，7例（16.3％）患者合并≥2种其他病原体感染。最常见的为细菌感染（21例，其中耐药菌感染6例）。详见表2。

**表2:** 43例血液病患者异基因造血干细胞移植后呼吸道合胞病毒（RSV）感染及治疗情况

临床资料	结果
感染部位［例（%）］	
上呼吸道感染	18（41.9）
下呼吸道感染	25（58.1）
混合感染［例（%）］	
甲型流感病毒	3（7.0）
乙型流感病毒	3（7.0）
腺病毒	3（7.0）
肺炎支原体	1（2.3）
巨细胞病毒	3（7.0）
EB病毒	6（14.0）
细菌	21（48.8）
真菌	4（9.3）
感染RSV距移植间隔时间［d，*M*（范围）］	150（1~2 121）
感染RSV时血常规［×10^9^/L，*M*（范围）］	
WBC	3.33（0.15~12.30）
ANC	1.90（0.00~8.23）
淋巴细胞计数	0.71（0.02~4.90）
PLT	55.0（8.0~348.0）
免疫抑制剂［例（%）］	
钙调磷酸酶抑制剂	26（60.5）
其他	4（9.3）
症状［例（%）］	
低氧血症	20（46.5）
呼吸衰竭	8（18.6）
重症肺炎	7（16.3）
转ICU继续治疗［例（%）］	6（14.0）
气管插管辅助通气［例（%）］	4（9.3）
治疗［例（%）］	
利巴韦林	22（51.2）
静脉	5（11.6）
口服	12（27.9）
雾化吸入	10（23.3）
糖皮质激素	24（55.8）
静脉注射免疫球蛋白	17（39.5）

**注** ICU：重症监护室

发生RSV感染时患者中位WBC为3.33（0.15～12.30）×10^9^/L，中位ANC为1.90（0.00～8.23）×10^9^/L，中位淋巴细胞计数为0.71（0.02～4.90）×10^9^/L，中位PLT为55.0（8.0～348.0）×10^9^/L。

30例患者在感染RSV时正在接受免疫抑制剂治疗，其中使用环孢素A单药26例，其他免疫抑制剂4例。共22例患者接受利巴韦林治疗（口服给药10例，雾化吸入6例，静脉给药1例，静脉给药联合雾化吸入3例，静脉给药联合口服给药1例，口服给药联合雾化吸入1例）。5例患者因合并流感病毒感染而联合奥司他韦、玛巴洛沙韦或帕拉米韦治疗。24例（55.8％）患者由于合并GVHD、重症肺炎等使用糖皮质激素治疗。17例患者使用静脉注射免疫球蛋白治疗。

20例（46.5％）患者因RSV感染或合并其他感染引起低氧血症，6例（14.0％）转ICU治疗，4例（9.3％）行气管插管辅助通气，7例（16.3％）患者死亡。

患者RSV感染和治疗情况见表2。

三、RSV感染患者转归情况及死亡患者特点

截至2023年1月31日，本组患者中7例死亡。其中5例感染RSV时原发病为血液学复发状态，4例伴有耐药细菌感染，2例死于急性脑梗死。

死亡与生存两组患者性别、年龄、诊断、植入时间等差异无统计学意义（表3）。死亡患者感染RSV时PLT较低［15.0（10.0～62.0）×10^9^/L对74.5（8.0～348.0）×10^9^/L对，*P*＝0.003］，合并细菌感染比例较高［85.7％（6/7）对41.76％（15/36），*P*＝0.046］，血液学复发比例较高［71.4％（5/7）对13.9％（5/36），*P*＝0.004］。

**表3:** 异基因造血干细胞移植后呼吸道合胞病毒（RSV）感染生存与死亡患者临床资料比较

临床资料	生存组（36例）	死亡组（7例）	*P*值
女性［例（%）］	16（44.4）	1（14.3）	0.215
移植时年龄［岁，*M*（范围）］	39（21~66）	49（31~59）	0.177
临床诊断［例（%）］			0.059
急性髓系白血病	19（52.8）	6（85.7）	
急性淋巴细胞白血病	9（25.0）	0（0）	
急性混合型白血病	1（2.8）	0（0）	
骨髓增生异常综合征	6（16.7）	0（0）	
再生障碍性贫血	0（0）	1（14.3）	
淋巴瘤	1（2.8）	0（0）	
感染时血常规［×10^9^/L，*M*（范围）］			
WBC	3.53（1.10~12.30）	2.50（0.15~8.78）	0.439
ANC	2.05（0.00~7.90）	1.03（0.07~8.23）	0.152
淋巴细胞计数	0.71（0.02~4.90）	0.70（0.03~3.24）	0.489
PLT	74.5（8.0~348.0）	15.0（10.0~62.0）	0.003
混合感染［例（%）］			
甲型流感病毒	1（2.8）	2（28.6）	0.064
乙型流感病毒	2（5.6）	1（14.3）	0.064
腺病毒	3（8.3）	0（0）	0.579
肺炎支原体	1（2.8）	0（0）	0.837
巨细胞病毒	2（5.6）	1（14.3）	0.421
EB病毒	5（13.9）	1（14.3）	0.681
细菌	15（41.7）	6（85.7）	0.046
真菌	3（8.3）	1（14.3）	0.523
HCT-CI评分［例（%）］			0.736
0分	25（69.4）	4（57.1）	
1~2分	6（16.7）	3（42.9）	
≥3分	5（13.9）	0（0）	
供患者血型［例（%）］			0.698
相合	16（44.4）	3（42.9）	
主要不合	9（25.0）	3（42.9）	
次要不合	8（22.2）	1（14.3）	
主要不合+次要不合	3（8.3）	0（0）	
供者类型［例（%）］			0.649
单倍体	28（77.8）	5（71.4）	
同胞全相合	6（16.7）	2（28.6）	
无关	2（5.6）	0（0）	
干细胞来源［例（%）］			0.061
外周血	34（94.4）	6（85.7）	
骨髓+外周血	0（0）	1（14.3）	
骨髓+外周血+脐带血	2（5.6）	0（0）	
外周血MNC［×10^8^/kg，*M*（范围）］	9.85（5.90~17.50）	8.68（6.11~11.70）	0.669
外周血CD34^+^细胞（×10^6^/kg）	3.49（1.12~10.47）	3.06（2.12~4.51）	0.554
粒细胞植入时间［d，*M*（范围）］	13（8~38）	12（10~42）	0.629
血小板植入时间［d，*M*（范围）］	13（8~38）	12（10~42）	0.709
感染RSV时疾病状态［例（%）］			
完全缓解	25（69.4）	2（28.6）	0.042
血液学复发	5（13.9）	5（71.4）	0.004
分子生物学复发	6（16.7）	0（0）	0.319
RSV感染距移植间隔时间［d，*M*（范围）］	153（0~2 121）	131（6~507）	0.948
感染RSV时急性GVHD［例（%）］			0.096
无	32（88.9）	4（57.1）	
Ⅰ度	4（11.1）	3（42.9）	
Ⅲ/Ⅳ度	0（0）	0（0）	
感染RSV时慢性GVHD［例（%）］			0.700
无	30（83.3）	6（85.7）	
轻度	3（8.3）	0（0）	
中度	2（5.6）	1（14.3）	
重度	1（2.8）	0（0）	
感染RSV时免疫抑制剂［例（%）］			
钙调磷酸酶抑制剂	13（36.1）	4（57.1）	0.265
其他	3（8.3）	2（28.6）	0.180
症状［例（%）］			
低氧血症	15（41.7）	5（71.4）	0.222
呼吸衰竭	4（11.1）	4（57.1）	0.015
重症肺炎	3（8.3）	4（57.1）	0.008
转ICU［例（%）］	2（5.6）	4（57.1）	0.004
气管插管辅助通气［例（%）］	1（2.8）	3（42.9）	0.010
治疗［例（%）］			
利巴韦林	18（50.0）	4（57.1）	0.527
静脉	3（8.3）	2（28.6）	0.180
口服	11（30.6）	1（14.3）	0.652
雾化吸入	8（22.2）	2（28.6）	0.656
糖皮质激素	19（52.8）	5（71.4）	0.437
静脉注射免疫球蛋白	14（38.9）	3（42.9）	0.581

**注** HCT-CI：造血干细胞移植合并症指数；MNC：单个核细胞；GVHD：移植物抗宿主病；ICU：重症监护室

采用二项Logistic回归分析患者与死亡相关的因素（表4）。血液学复发（*OR*＝15.500，95％*CI* 2.336～102.848，*P*＝0.005）、甲型流感病毒感染（*OR*＝14.000，95％*CI* 1.064～184.182, *P*＝0.045）、感染RSV时PLT（*OR*＝0.945，95％*CI* 0.894～0.999，*P*＝0.048）与死亡相关，是移植后患者死亡的危险因素。

**表4:** 血液病患者造血干细胞移植后呼吸道合胞病毒（RSV）感染死亡危险因素分析

因素	*B*	*OR*（95%*CI*）	*P*值
血液学复发	2.741	15.500（2.336~102.848）	0.005
合并甲型流感病毒感染	2.639	14.000（1.064~184.182）	0.045
感染RSV时血小板计数	−0.057	0.945（0.894~0.999）	0.048
年龄	0.046	1.047（0.976~1.122）	0.200
感染RSV距移植间隔时间	−0.001	0.999（0.996~1.002）	0.622
细菌感染	2.128	8.400（0.914~77.208）	0.060
感染RSV时WBC<2×10^9^/L	−0.195	0.832（0.543~1.246）	0.357
感染RSV时淋巴细胞计数<0.1×10^9^/L	−0.165	0.848（0.348~2.067）	0.716
合并Ⅱ度急性GVHD	1.917	6.800（0.774~59.746）	0.084
应用糖皮质激素	0.805	2.237（0.383~13.074）	0.371

**注** *B*：回归系数；*OR*：比值比；95％*CI*：95％置信区间；GVHD：移植物抗宿主病

## 讨论

RSV是一种包膜病毒，具有非分段、单链、负义RNA基因组，其感染具有明显的季节性。本研究43例患者中，仅2例于2023年10月感染，其余41例均在2023年12月至2024年1月之间感染，符合北半球国家冬季高发RSV感染的规律。有研究认为，因COVID-19全球大流行而实施的限制性措施使过去两年RSV感染减少，由于限制性措施对人口产生过度保护，人群中RSV特异性抗体随之下降[14]；限制性措施废除后，可能出现更为广泛和严重的RSV流行[15]。2022年，已观察到儿童和成人RSV感染率增加（包括典型季节性之外的流行）[16]。随着RSV在健康人群中的流行，移植后患者更容易在社区通过呼吸道飞沫传播感染RSV。

既往研究显示，移植后6个月内、白细胞计数≤2.0×10^9^/L、淋巴细胞绝对计数≤0.1×10^9^/L、合并≥Ⅱ度急性GVHD、大剂量糖皮质激素治疗、吸氧治疗/机械通气、预处理方案含ATG、存在肺部基础疾病与RSV感染患者死亡风险增加有关[17]–[19]。本研究随访时间较短，大多数患者RSV感染发生于移植后30 d以内，采用二元Logistic回归分析发现血液学复发、合并甲型流感病毒感染、感染RSV时血小板减少是移植后患者死亡的危险因素。

本研究中，10例患者感染RSV时为血液学复发状态，其中5例死亡（1例患者血液学复发后放弃治疗死亡，其余4例因急性脑梗死、合并重症肺炎等死亡）。移植后复发和感染是血液系统疾病患者死亡的重要原因，复发患者可能存在免疫功能通路失调引起免疫逃逸[20]和免疫重建异常[21]，更易发生感染。复发患者在RSV感染治疗的选择上也存在问题。尽管目前利巴韦林是成人RSV感染的主要治疗药物，但不同给药方式产生的疗效均缺乏明确证据，并且利巴韦林的血液学不良反应会给复发患者带来更多治疗负担。

在本研究中，55.8％的患者感染RSV时正在接受糖皮质激素治疗，提示患者感染RSV时处于免疫功能低下状态。本组病例感染RSV的中位时间为移植后150 d，大多数患者在感染RSV时粒细胞、血小板已成功植入，但移植后患者需要12～18个月逐渐完成免疫重建，在此期间仍有较高的感染风险。本研究中，高达62.8％（27例）的患者存在混合感染，其中细菌感染最为常见，并且死亡患者合并细菌感染比例更高。一项针对免疫系统功能低下患者的回顾性队列研究显示，合并细菌感染并引起肺炎能够造成高达80％的RSV相关死亡[22]。合并细菌感染使发展为LRTI的风险增高，这可能是由于RSV引起呼吸道上皮损伤而增加细菌黏附[23]–[24]。本研究中3例患者合并甲型流感病毒感染，2例死亡，其中1例同时合并耐药细菌感染，1例同时合并乙型流感病毒感染，2例患者均因重症肺炎死亡。由于本研究样本量较小，尚需扩大样本量验证结果。

MD Anderson Cancer Center提出的移植后患者RSV感染危险因素分层将移植后30 d内感染RSV或诊断RSV感染时未完成造血重建作为一项危险因素[25]。本研究中，4例患者感染RSV时血小板未植入，其中2例存在LRTI，1例转入ICU并行插管呼吸机辅助通气治疗后病情好转，1例经抗病毒等治疗后RSV转阴。1例患者血小板重建不良，RSV感染25 d后因脑梗死、缺血性心肌病、血栓性微血管病（TMA）死亡。本研究中，死亡患者感染RSV时PLT明显低于生存患者，且中位PLT<20×10^9^/L，提示血小板减少可能在病毒感染中起到一定作用，但仍需进一步探究。

本研究共纳入43例患者，25例发生LRTI，病死率为16.3％（7/25），高于既往研究[10],[22]。但本研究并未在所有移植后患者中开展呼吸道病原筛查，而是纳入了allo-HSCT患者，并在患者出现呼吸道症状或出现发热时检测呼吸道病原，这说明本研究纳入的患者临床表现更重，且RSV检出率相对较高。考虑到患者的治疗意愿，部分患者未在适当时机转入ICU积极治疗或行气管插管改善通气，本组病例病死率较高可能与此有关。

综上所述，本研究结果初步显示移植后RSV感染患者预后不良，血液学复发、感染RSV时血小板计数较低、合并急性GVHD与死亡相关关。本研究样本量较小，为单中心回顾性描述性研究且缺少下呼吸道标本，以上结论尚需前瞻性队列研究验证。
